# Bilateral Facial Palsy: A Case Study of an Exceedingly Rare and Difficult Diagnosis

**DOI:** 10.7759/cureus.18900

**Published:** 2021-10-19

**Authors:** Gaurav Jha, Sabeen Azhar, Shivani Kuttuva, Sameel Elahi, Asad Baseer

**Affiliations:** 1 Neuroscience, Queen's Hospital, Barking, Havering and Redbridge University Hospitals NHS Trust, London, GBR; 2 Acute Medicine, Queen's Hospital, Barking, Havering and Redbridge University Hospitals NHS Trust, London, GBR; 3 Surgery, Royal Vicoria Infirmary, Newcastle upon Tyne Hospitals NHS Foundation Trust, Newcastle, GBR; 4 Acute Medicine, Princess Alexandra Hospital, The Princess Alexandra Hospital NHS Trust, Harlow, GBR; 5 Orthopaedics, Leicester Royal infirmary, University Hospitals of Leicester NHS Trust, Leicester, GBR

**Keywords:** bell’s palsy, bilateral facial palsy, heerfordt, sarcoidosis

## Abstract

Bilateral facial nerve palsy is a rare condition, representing only 0.3-2.0% of all facial palsy cases. Facial paralysis constitutes the result of a diverse array of systemic disorders and heterogeneous aetiologies and thus represents a diagnostic challenge. This case report describes a previously healthy male who presented to the emergency department numerous times within a few weeks with unrelated non-specific symptoms. These symptoms could not be attributed to any specific aetiology after various radiological and laboratory examinations, and hence presented a diagnostic dilemma until he developed bilateral seventh nerve palsy and was admitted for a further workup.

## Introduction

Unilateral facial palsy is a fairly common diagnosis with an incidence of 20-25 per 100,000 people. Most instances are linked to Bell's (i.e., idiopathic) palsy, accounting for more than 50% of these cases [1,2]. Bilateral facial paralysis, as opposed to unilateral facial paralysis, is exceedingly rare and accounts for only 0.3-2% of cases presenting with facial palsy [3]. The incidence of such cases is approximately one per five million per year [4]. Bilateral facial palsy is characterised by facial paralysis on both sides of the face, with the onset of symptoms on both sides either simultaneously or the second side being affected within 30 days of the first side [5]. Most of these patients have significant underlying medical problems and might exhibit bilateral facial paralysis as the first sign. Therefore, we recommend conducting a thorough investigation using both laboratory and radiological evidence to evaluate all instances of bilateral facial palsy.

## Case presentation

A 32-year-old Asian male presented to the emergency department with complaints of right-sided facial weakness, perioral numbness, speech difficulty, and altered taste sensation, which had started five days ago. Interestingly, he had been seen in the emergency department multiple times before this attendance and sent home with an outpatient neurology follow-up as the investigation results had been within the standard parameters. The patient also gave a history of a flu-like illness two weeks earlier. He had a past medical history of obstructive sleep apnea and bipolar disorder, and his regular medications included lithium carbonate and sodium valproate. He was a non-smoker and denied any alcohol or drug use.

**Figure 1:**
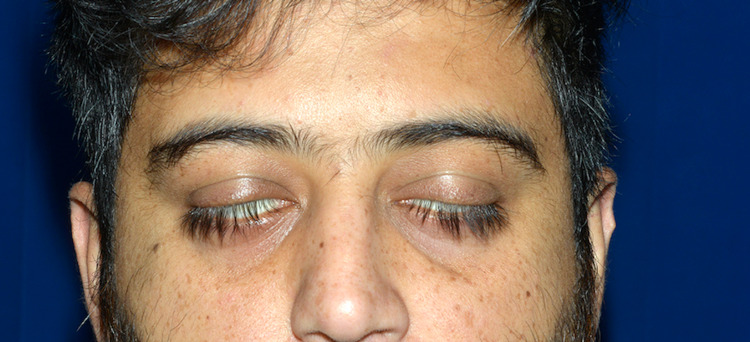
Image showing inability to close both eyes

**Figure 2:**
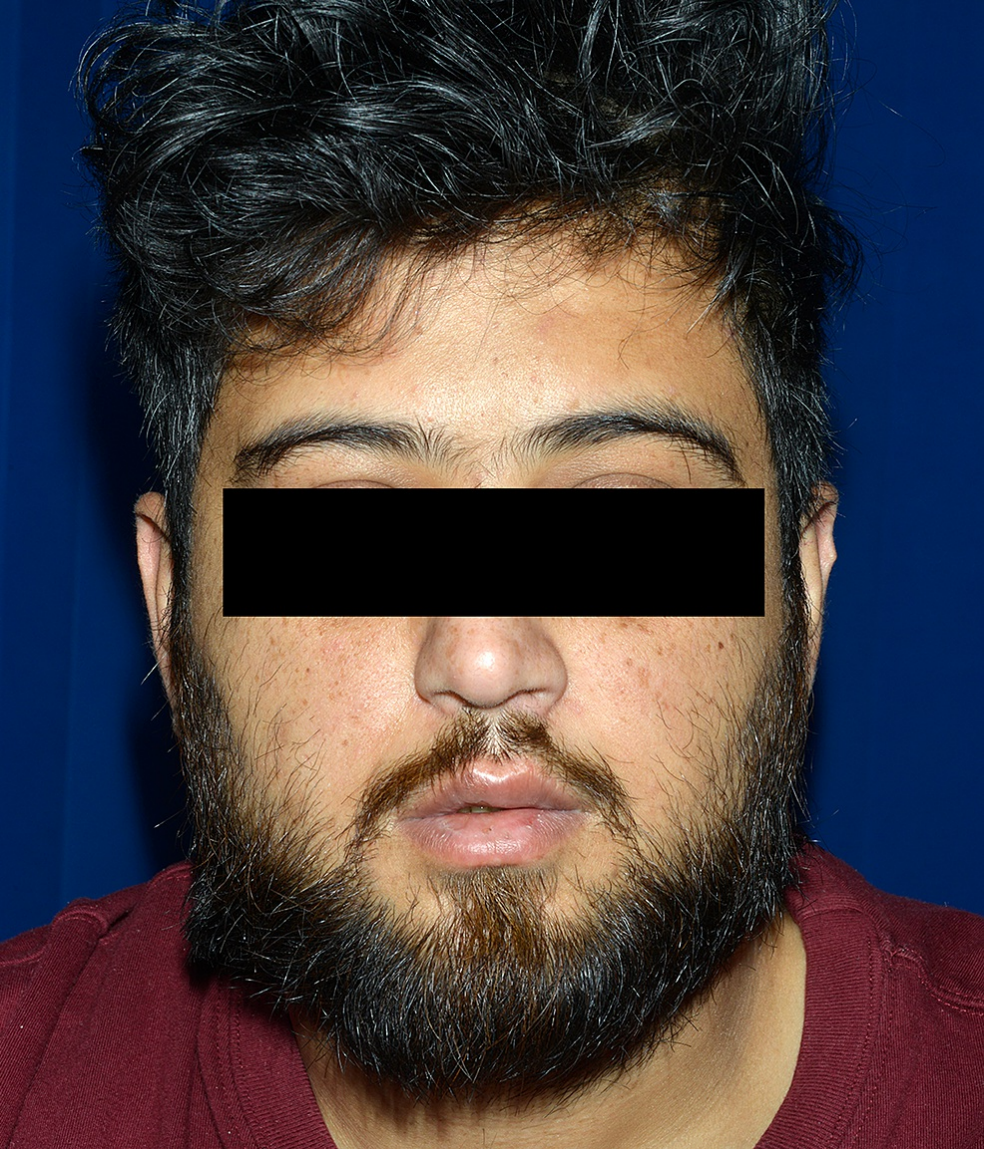
Image showing an attempt to raise eyebrows

**Figure 3:**
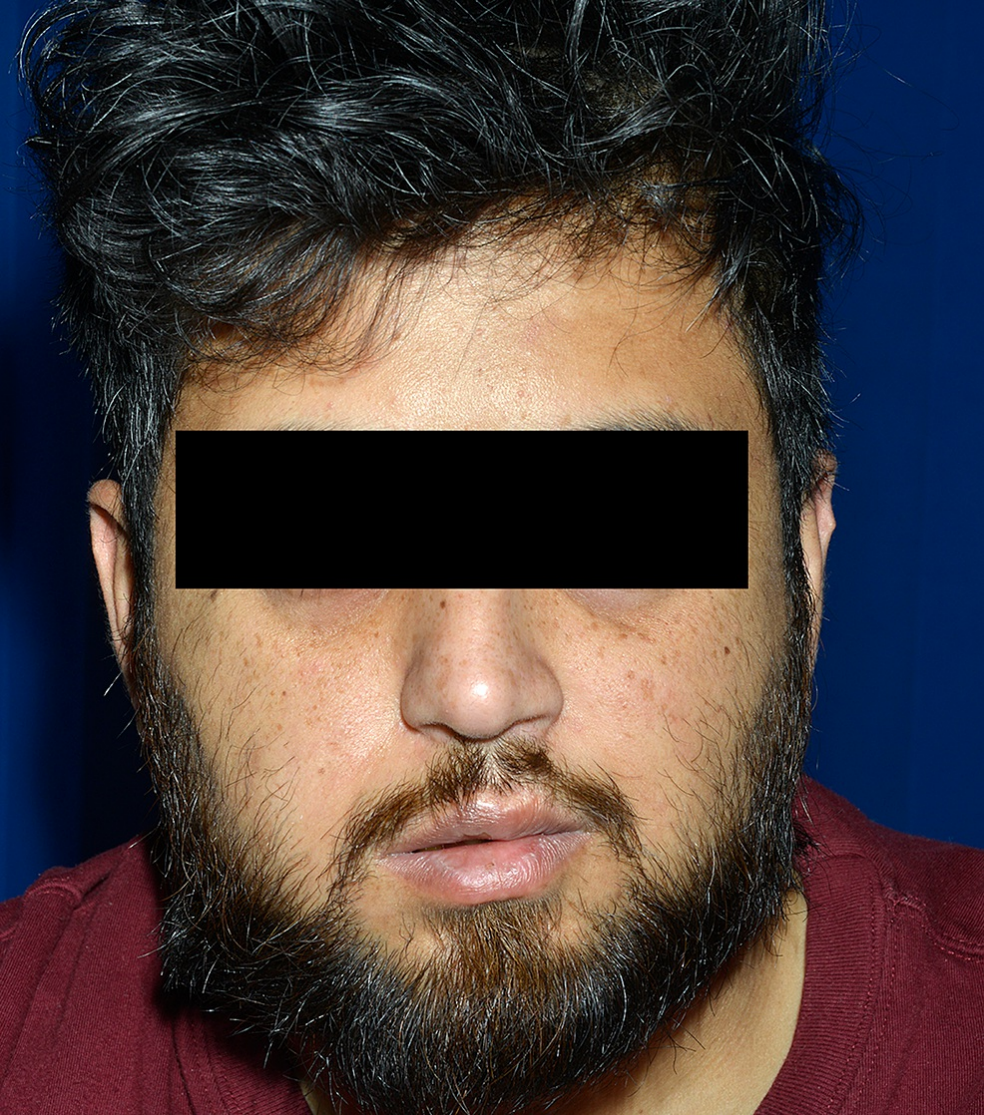
Image showing an attempt to blow the cheeks

The patient had been referred to be assessed by the stroke team. His symptoms had started with right-sided facial weakness, eventually progressing to the left side. He denied any history of trauma, rashes, travel abroad, or tick bites. On examination, he had a bilateral facial weakness with the inability to elevate eyebrows, puff out cheeks, or smile, and inadequate closure of the eyes (Figures 1-3). The neck was supple, and neurological examination revealed mild facial sensory deficits with bilateral lower motor neuron palsy. The rest of the neurological examination revealed no abnormalities. A complete blood count, comprehensive metabolic panel, and CT scan of the head did not reveal any significant findings. After a thorough exam, the stroke team ruled out a stroke, and the patient was handed over to the medical team for further workup.

The neurology team advised a lumbar puncture to rule out infection before commencing any steroids. After two failed lumbar punctures in the lateral position due to increased basal metabolic index (BMI), a lumbar puncture was attempted in the sitting position, and cerebrospinal fluid (CSF) analysis showed the following results: glucose: 3.0, WBC: 6, lymphocytes: 6, RBC: <1, and protein: 0.75; Gram stain was negative. A CSF oligoclonal band was not detected. Electromyography (EMG) showed partial right facial nerve lesion, demyelinating in nature, and no signs of acute motor axonal loss and left facial lesions. Subsequent MRI head with contrast was unremarkable. No paraproteins were detected on serum electrophoresis, and the immunology, vasculitis, and viral screen were negative (Table 1).

**Table 1:** Laboratory test results FBC: full blood count; Hb: hemoglobin; WBC: white blood cell; RBC: red blood cell; HCT: hematocrit; PLT: platelets; ESR: erythrocyte sedimentation rate; PT: prothrombin time; APTT: activated partial thromboplastin time; ALP: alkaline phosphatase; ALT: alanine aminotransferase; GGT: gamma-glutamyl transferase; HDL: high-density lipoproteins; LDL: low-density lipoprotein; CRP: C-reactive protein; ACE: angiotensin-converting enzyme; CSF: cerebrospinal fluid; Ach: acetylcholine; HIV: human immunodeficiency virus; HSV: herpes simplex virus; VZV: varicella-zoster virus; DNA: deoxyribonucleic acid; RNA: ribonucleic acid; HBsAg: hepatitis B surface antigen; EIA: enzyme immunoassay; EBV: Epstein-Barr virus, PCR: polymerase chain reaction; Ab: antibody; IgG: immunoglobulin G; IgM: immunoglobulin M

Test	Value	Reference range	Units
FBC			
Hb	125	130–180	g/L
WBC	10.5	4–11	10^9^/L
RBC	4.58	4.5–6.5	10^12^/L
HCT	0.37	0.40–0.52	L/L
PLT	225	150–450	10^9^/L
Neutrophils	8.3	2.0–7.5	10^9^/L
Lymphocytes	1.7	1.5–4.5	10^9^/L
Monocytes	0.5	0.2–0.8	10^9^/L
Eosinophils	0.0	0–0.4	10^9^/L
Basophils	0.0	0–0.1	10^9^/L
ESR	20	1–10	mm/hr
PT	10.8	10.0–13.0	Seconds
APTT	21.6	23–30	Seconds
Serum B12	983	180–900	ng/l
Serum folate	5.9	3–20.0	μg/L
Ferritin	255	20–300	μg/L
Biochemistry			
Sodium	142	133–146	mmol/L
Potassium	5.0	3.5–5.3	mmol/L
Urea	3.7	2.5–7.8	mmol/L
Creatinine	70	59–104	mmol/L
ALP	64	30–130	U/L
ALT	56	10–50	U/L
Calcium	2.32	2.2–2.6	mmol/L
Phosphate	1.25	0.8–1.5	mmol/L
GGT	22	0–60	U/L
Amylase	121	28–100	U/L
Cholesterol	4.8	0–5.0	mmol/L
Triglycerides	1.36	0–1.7	mmol/L
HDL cholesterol	0.82	1.2–3.0	mmol/L
LDL cholesterol	4.8	1.0–3.0	mmol/L
Glucose	8.9	<5.5	mmol/L
CRP	11	<5	mg/L
ACE	48.4	8–52	IU/L
CSF analysis			
CSF glucose	3.0	50–80	mg/100mL
CSF protein	0.30	0.15–0.45	g/L
CSF oligoclonal band	Negative		
CSF viral PCR screen	Not detected		
WBCs	6	0–8	mm^3^
RBCs	<1	<1	mm^3^
Gram stain	No organism seen		
Culture	No growth after 48h incubation		
Ach. Receptor abs	0.34	<0.5	nmol/L
Serology			
HIV 1,2 Ab p24 Ag	Not detected		
HSV 1 DNA PCR	DNA not detected		
HSV 2 DNA PCR	DNA not detected		
VZV DNA (qualitative)	DNA not detected		
HBsAg (EIA)	Not detected		
EBV screen	Not detected		
Enterovirus RNA PCR	RNA not detected		
Treponemal Ab	Not detected		
B. burgdorferi IgG/IgM	Negative, no serological evidence of B. burgdorferi		

More information came to light during a thorough and detailed history taking. Family history revealed that both the patient's mother and sister had bilateral facial palsy, and his mother had been diagnosed with sarcoidosis. At this point, serum angiotensin-converting enzyme (ACE) level was requested due to the suspicion of sarcoidosis and was found to be within the range. Chest radiograph showed no hilar adenopathy. A CSF ACE level was not requested, and hence sarcoidosis could not be completely ruled out. An eye examination conducted to check for ocular manifestations revealed no abnormalities. On day six of admission, the patient was commenced on oral steroids after a central nervous system (CNS) infection was ruled out. By this time, there had been a complete resolution of the left-sided facial weakness and the initial complaint of the neck pain; however, the right-sided facial palsy and the pain around the ears were still present. Otolaryngological examination revealed tenderness in the parotids and submandibular region even though the glands were not enlarged. Hence, a clinical diagnosis of Heerfordt syndrome was considered. The patient was advised to complete a 10-day course of steroids and sent home with eye patches once his symptoms were found to be improving. Follow-up at one week showed improvement in right-sided facial weakness as well. Slurring of speech became better but did not revert to baseline. The patient has developed no additional symptoms and is currently under outpatient neurology follow-up.

## Discussion

A significant difficulty for clinicians in numerous specialities and care settings is determining the appropriate triage of patients with acute facial paralysis. Bilateral facial palsy is often a manifestation of a severe underlying systemic disease and warrants urgent medical intervention, unlike unilateral presentation, which is usually secondary to idiopathic Bell's palsy and is often not investigated further. A recent review identified a misdiagnosis rate of <1% in the emergency setting, but previous literature has suggested rates as high as 20%, with 5% of lower motor facial paralysis being attributed to tumours [6]. The aetiologies of bilateral facial palsy can include congenital, infectious, vascular, metabolic, autoimmune, neoplastic, neurological, or traumatic causes [7,8]. It is crucial to identify the underlying cause because treatment and prognosis depend on it. After the initial blood-work, additional investigations should be guided by a detailed history and physical examination. Symptom characterisation (e.g., time of onset), associated symptoms (diplopia, facial numbness, otological symptoms, aphasia), relevant risk factors (e.g., viral prodrome, immunisation), and prior palsy history can give us clues towards the diagnosis. In our case of bilateral facial nerve palsy, the patient did provide an account of flu-like illness two weeks before this presentation. However, no consideration was given to antiviral treatment, which is a retrospective thing because antivirals can be considered in cases with a strong history of viral prodrome. The American Academy of Neurology (AAN) and American Academy of Otolaryngology-Head and Neck Surgery Foundation (AAO-HNSF) have recommended administration of oral steroids (prednisone 50-60 mg daily) and, less strongly, antiviral medications (acyclovir or valacyclovir) to patients with new (defined as <72 hours in onset by AAO-HNSF) Bell's palsy [9]. A reasonable approach is to start by ruling out potentially life-threatening causes like Guillain-Barré syndrome (GBS) by looking for peripheral areflexia and liquoric dissociation on a CSF analysis as GBS presenting with facial nerve palsy is fatal in up to 50% of the cases [10]. It is equally important to rule out intrapontine and prepontine tumours along with traumatic fractures by doing a CT or preferably an MRI of the brain [11]. In our case of bilateral facial nerve palsy, CSF analysis was within normal parameters, and brain imaging revealed no abnormalities. Bilateral facial nerve palsy that develops rapidly, like in our case over a few days, suggested more of an infectious or autoimmune aetiology, especially in a setting with no history of trauma. Moreover, synchronous palsy with the involvement of the contralateral side within 30 days also pointed towards a systemic disease rather than a localised inflammatory condition [12]. Lyme disease is the most common among other infectious causes of bilateral facial nerve palsy (30%-35%) and was ruled out by negative serology [13]. It was also deemed unlikely as our patient denied any recent travel to endemic areas or contact with ticks. An acute Epstein-Barr virus (EBV) infection was excluded by a negative Epstein-Barr virus capsid antigen (VCA) IgM antibody test. Other common systemic causes include diabetes, which was ruled out by normal blood sugar levels. Interestingly, although the history of symptom onset was suggestive of autoimmune aetiologies like sarcoidosis and amyloidosis, no initial workup was done to explore these causes. Sarcoidosis was not deemed a possible cause until it was discovered that the patient's mother had sarcoidosis and complained of bilateral parotid pain. At this point, serum ACE levels were requested, and a chest X-ray was ordered to exclude hilar adenopathy. Sarcoidosis is a multi-system granulomatous disorder of unknown aetiology, which most commonly affects the pulmonary system. Neurological manifestations include cranial neuropathies, the most frequently affected being the facial nerve (unilateral palsy in 1-3% and bilateral palsy in 0.1-0.2% of cases) [14]. Diagnosis is supported by serum ACE levels, tissue biopsy of affected organs, and enlarged lymph nodes on a chest CT. With no apparent systemic involvement, a biopsy was not indicated. However, it is worth considering whether a CT chest should have been requested as an initial workup. Glucocorticoids for two weeks are indicated in the treatment of bilateral facial nerve palsy if sarcoidosis is the suspected or confirmed cause. Even though steroid treatment was initiated in our case, it is worth noting that it was commenced on day six of admission when the left-sided weakness had started to improve. Eventually, the right-sided weakness improved as well but did not resolve completely. Although there is no established association between the time of initiation of steroids and prognosis, this case nevertheless highlights the importance of early consideration by the physician for the use of empiric corticosteroids given their efficacy in numerous differential diagnoses. Early neurology consultation in these cases ensures such decisions are not delayed. Thus, in our case, although our patient was eventually discharged with the clinical diagnosis of Heerfordt syndrome, it was a diagnostic dilemma as investigations could not confirm it. Heerfordt syndrome is an acute and rare presentation of sarcoidosis. It manifests as fever, uveitis, and bilateral parotid swelling with bilateral facial palsy. However, the typical presentation of this syndrome is rare and is seen in 0.3-1.2% of cases of sarcoidosis [15]. Very rarely, bilateral facial nerve palsy can present as an initial isolated manifestation of sarcoidosis. It would be interesting to follow these patients to see if they develop sarcoidosis later on in life.

## Conclusions

Bilateral facial nerve palsy is an uncommon and diagnostically challenging presentation. Our case report highlights the importance and need for a thorough history taking, including family history, physical examination, and investigating the full spectrum of possible diagnoses in all instances presenting with bilateral facial nerve palsy instead of going in with a tunnel vision. Bilateral facial paralysis has a variety of causes, and the manifestation of Heerfordt syndrome is variable. Hence, a complete understanding of this condition can assist the clinician in making a proper and accurate diagnosis. While management varies by aetiology, the physician should consider early empiric corticosteroids given their efficacy in numerous differential diagnoses.
